# Optimal Suture Bite Size for Closure of Feline Linea Alba—A Cadaveric Study

**DOI:** 10.3389/fvets.2019.00441

**Published:** 2019-12-10

**Authors:** Amanda L. Bartier, Aylin Atilla, Rebecca Archer, Grace P. S. Kwong

**Affiliations:** Faculty of Veterinary Medicine, University of Calgary, Calgary, AB, Canada

**Keywords:** feline, linea alba, bite size, suture, cat, SBSI

## Abstract

**Objective:** This study aimed to determine the most appropriate suture bite and stitch interval (SBSI) size for closing feline linea alba.

**Study design:** Randomized *ex vivo* mechanical testing.

**Sample population:** Ventral abdominal walls from 35 male feline cadavers were harvested and separated into 125 segments.

**Methods:** Segments were incised along midline then sutured back together using 3-0 polydioxanone using one of the following SBSI: 3, 5, 7.5, or 10 mm whereby the distance represents the distance both between the suture bites, and from the bite to incision line. The location of segments as well as the weight of the cadaver were recorded. A single linear distraction mechanical breaking test was performed. Statistical analyses (logistic and linear regression) were performed to determine which factors were associated with visual and mechanical failure, as well as load at failure or maximum displacement.

**Results:** SBSI was significantly associated with load at failure or maximum displacement (*p* < 0.001). In particular, 5 mm SBSI had the highest load at failure amongst all the bite sizes (LSmeans = 27.55N, 95% Confidence Interval (CI): 23.65–31.44); this was significantly higher than 7.5 mm (LSmeans = 19.15N, 95% CI: 15.26–23.05, *p* = 0.016) and 10 mm (LSmeans = 16.55N, 95%CI:12.39–20.70, *p* = 0.0012) but not significantly higher than 3 mm (LSmeans = 23.78N, 95% CI: 19.69–27.86, *p* = 0.2). Increased SBSI increased the odds of visual failure (*p* < 0.001) whereas increased weight of the cadaver reduced the odds of visual failure (OR = 0.52, 95%CI: 0.30–0.88, *p* = 0.016).

**Conclusion:** The 5 mm SBSI had the highest load at failure and was not significantly different from the 3 mm SBSI when apposing feline linea alba using 3-0 polydioxanone.

**Clinical significance:** Using 5 mm SBSI is the preferred bite size compared to 7.5 or 10 mm SBSI when apposing feline linea alba with 3-0 polydioxanone.

## Introduction

Abdominal surgeries in feline patients represent one of the most common surgeries performed in the species (1). Current recommendations for closing feline linea alba are primarily based on human, equine, and canine studies (2–5). Recommendations for bite spacing from each other and from the linea edge range from 5 mm (6) in porcine fascia, to 10 mm (5)−15 mm (7) in humans. More recent studies indicate that sutures placed closer to the incision line are associated with reduced development of necrotic tissue (8) and increased wound strength (9) in humans. There is little data available regarding the incidence of dehiscence and herniation in cats following abdominal surgery. One study reported 1 dehiscence in 550 abdominal surgeries of dogs and cats (10), and another reported 1 dehiscence in 66 abdominal surgeries (3). In human patients, the risk of dehiscence is estimated to be 2% (11), and herniation is as high as 10 to 20% (6, 11). We do not have a good grasp on the incidence of true incisional herniation in veterinary medicine. It is currently unknown if the commonly recommended suturing parameters are suitable for feline linea alba closure, as there may be differences in the mechanical properties between human, equid, canine, and feline fascia. One recent biomechanical study demonstrated that 2 mm bite sizes was ideal for feline linea alba closure, but their method of calculating bite size did not take into account the entire width of fascia incorporated in the suture bite (12).

This experimental study aimed to determine an effective protocol for suturing feline linea alba focusing on the most appropriate suture bite and stitch intervals (SBSI).

We hypothesized that the smallest SBSI of 3 mm would have the highest load to failure compared to larger SBSI sizes.

## Materials and Methods

This study was performed in accordance with Institutional Animal Care And Use guidelines and was approved by the Institutional Animal Care And Use Committee at the University of Calgary, Faculty of Veterinary Medicine. Thirty-five feline abdominal walls, between 11 and 19 cm long, were harvested from male (castrated or intact) cadavers. Mature male cadavers were used because female cadavers are more likely to contain a scar from a previous ovariohysterectomy which would either have altered the study results or greatly diminished the amount of useable linea. Maturity was assessed by looking at body size and the presence of permanent dentition. The weight of the cat was measured and recorded. The technique for harvesting the ventral abdominal wall was performed as follows: one incision was made with a scalpel from the right to the left side of the abdomen at the level of the pubis, through the entire body wall. This incision extended laterally beyond the nipple line and then was extended cranially up to the last rib. This was repeated on the other side and then the incision was joined together from right to left at the level of the last rib. Once the ventral body wall was removed, the skin and subcutaneous tissue was separated from the external abdominal muscles using blunt dissection. The length of each abdominal wall was measured, then cut into segments that were 3.5 cm long. This was dictated by the largest size clamp available to use to test the body wall segments. Between 2 and 5 segments were obtained per cadaver. The segments were then separated into three groups as follows: Segments were marked as “umbilicus” if it contained the umbilicus. “Cranial” and “caudal” segments were cranial and caudal to the umbilicus, respectively. Each segment was stretched onto a piece of paper tissue, and placed into individually labeled bags with a small volume of 0.9% NaCl saline. The segments were frozen in a −18°C freezer until suturing.

The samples in each group (umbilicus, cranial, and caudal) were randomly distributed into one of the four SBSI size groups using a random number generator. The following SBSI sizes were tested: 3, 5, 7.5, and 10 mm, whereby the distance represents the distance both between the suture bites, and from the bite to incision line. Prior to suturing, samples were thawed at room temperature in a 0.9% NaCl bath. A number 10 scalpel was used to create an incision down the center of each segment, in the linea alba. The bites were measured with a ruler and marked using a black marker to ensure consistent and accurate suture placement. Using 3-0 polydioxanone on a taper SH needle, a simple continuous pattern was placed through the linea alba to approximate the incision edges including only the external rectus fascial layers. Suture placement was measured from the actual cut edge of fascia, not from the edge of the rectus. The samples were then refrozen at −18°C until they were tested. In total, the samples were frozen for <1 month.

Segments were kept frozen until just a few minutes prior to being tested when they were thawed in a physiologic saline bath. Each segment underwent a single breaking strength test. The segments were mounted into a unidirectional materials testing machine (Bose Electroforce 3200, provided by Zymetrix Biomaterials and Tissue Engineering TDC, University of Calgary), and the gripping device tightened at a rate of 10 mm/min for a total displacement of 12 mm (Supplementary Figure 1). A load-displacement curve was recorded once per sample. The largest clamp available for this machine was 3.5 cm long, and the samples were cut to this same length to ensure each segment experienced the same amount of force. Each tissue segment was loaded into the machine and tension was measured at 0 N prior to the start of each test. Visual failure was defined as visual distraction of the linea edges (Supplementary Figure 1), visible tearing of the fascia at the level of the suture line, suture breakage, or knot slippage. Whether obvious visual failure occurred was recorded for each sample and the force at which this happened. Mechanical failure was identified as the time point when a rapid drop in force on the load-displacement curve occurred. Whether mechanical failure occurred and the force at which this occurred was recorded for all samples. The tissue SBSIs were compared to determine which parameters withstood the highest force before failure, while accounting for the cadaver weight and location of each segment (cranial, umbilical, and caudal abdomen). Samples were prepared and tested by the same person.

Each step of the procedure was performed en bloc to ensure efficiency and consistency in sample treatment.

### Statistical Analysis

Logistic regression models were performed to determine which factors (sample location, SBSI, load, and cat weight) were associated with visual and mechanical failure. Linear regression models were performed to determined which factors (SBSI, location, or cat weight) was associated with force at failure. The load at mechanical failure or maximum displacement was compared using one way ANOVA. Power analysis was performed after experimentation and determined the minimum number of samples required for statistical significance using Fisher's Least Significant Difference (LSD) method with a power of 0.8 and alpha of 0.05. All analyses were performed using R software (13). Values of *P* < 0.05 were considered statistical significant for all tests.

## Results

### Descriptives

A total of 125 samples were randomly distributed into 12 test groups, as shown in Table 1. Out of the 125 samples used, the majority underwent both visual (80.8%) and mechanical failure (92.0%) (Table 2). The load at mechanical failure or maximum displacement was not significantly different between sample locations (*p* > 0.4 between groups) but varied with SBSIs (*p* < 0.001). The average (±standard deviation, SD) cadaver weight was 4.65 kg (±1.0) with a range of 2.70–6.38 kg.

**Table 1:** Total number of samples per test group.

**Test group (mm)**	**Cranial**	**Umbilical**	**Caudal**	**Total**
3	7	9	14	30
5	9	9	15	33
7.5	9	9	15	33
10	7	9	13	29
Total	32	36	57	125

**Table 2:** Number of samples that underwent mechanical and visual failure by bite size and location, and average load in *N* at point of failure or maximum displacement (*n* = 125).

	**Mechanical** ** failure (%)**		**Visual** ** failure (%)**		**Load *(N)* at mechanical failure or maximum displacement (Std Dev)**
	**No**	**Yes**	**No**	**Yes**	
Bite size					
3	3 (2.4)	27 (21.6)	14 (11.2)	16 (12.8)	23.8 (12.7)
5	3 (2.4)	30 (24.0)	7 (5.6)	26 (20.8)	27.5 (12.1)
7.5	4 (3.2)	29 (23.2)	3 (2.4)	30 (24.0)	19.2 (11.6)
10	1 (0.8)	28 (22.4)	1 (0.8)	28 (22.4)	16.5 (8.0)
Location					
Cranial	4 (3.2)	28 (22.4)	8 (6.4)	24 (19.2)	23.3 (13.7)
Umbilical	1 (0.8)	35 (28.0)	4 (3.2)	32 (25.6)	22.8 (10.7)
Caudal	6 (4.8)	51 (40.8)	13 (10.4)	44 (35.2)	20.5 (11.7)

### Risk of Mechanical Failure

**Figure 1:**
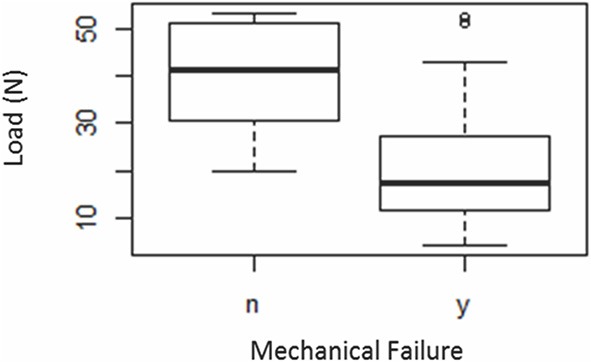
Box-plot representing the load at maximum displacement and load at mechanical failure (*n* = 125): “y”—failed, “n”—did not fail. The bold horizontal line within the boxes indicate the median load values. The circular symbols indicate outlying data points.

In the logistic regression model for risk of mechanical failure, the only significant factor was the load experienced by the sample (Table 3). For each unit increase in force there was a 0.87 [95% Confidence Interval (CI): 0.82–0.94] reduction in the odds of mechanical failure, *p* < 0.001. That is, if mechanical failure occurred, it occurred at a relatively lower load. The average (± SD) load at maximum displacement for samples that did not fail was 39.8 N (±11.6) and the average load for samples that did fail was 20.3 N (±10.7) (Figure 1).

**Table 3:** Logistic regression for mechanical failure (*n* = 125).

	**Estimate**	**Std error**	**Odds ratio**	***p*-value**
Intercept	6.46	1.31	640.07	<0.001
Load (*N*)	−0.13	0.04	0.87	<0.001

### Risk of Visual Failure

The most common visually identified form of failure was the tissue stretching, or the sutures tearing through the tissue. In the logistic regression model for risk of visual failure, the significant factors were the SBSI (*p* < 0.001) and the weight of the cadaver (*p* = 0.016) (Table 4). After adjusting for potential differences in weight, using a SBSI of 3 mm as the reference group, the odds ratio increased with increasing SBSI, with increased odds of 3.64, 9.07, and 25.59 of visual failure for SBSIs of 5, 7.5, and 10 mm, respectively. When SBSI was accounted for, every unit increase in weight reduced the odds of visual failure by 0.52 (95% CI: 0.30–0.88) (Supplementary Figure 1).

**Table 4:** Logistic regression for factors associated with visual failure (*n* = 125).

	**Estimate**	**Std error**	**Odds ratio**	***P*-value**
Intercept	3.27	1.32	26.42	0.01
Bite size				
3 mm bite	Ref	–	–	–
5 mm bite	1.29	0.60	3.64	0.030
7.5 mm bite	2.21	0.74	9.07	0.003
10 mm bite	3.24	1.10	25.59	0.003
Weight (kg)	−0.66	0.27	0.52	0.016

### Force at Failure

**Figure 2:**
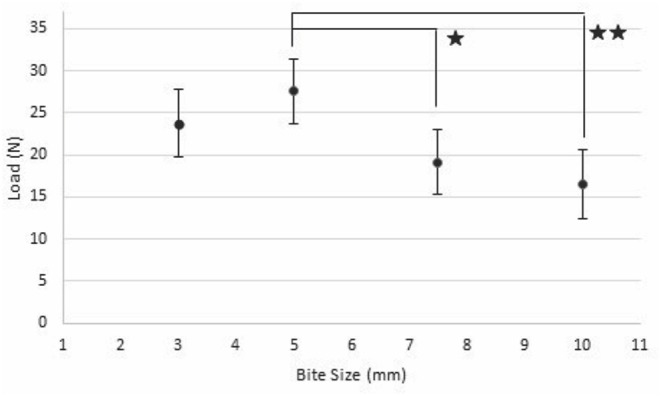
Expected load (black dot) with 95% CI (vertical bar) at failure or maximum displacement for each bite size and suture distance (x-axis: Bite Size) (*n* = 125). One star indicates groups are significantly different with *P* < 0.02, two stars indicates a significant difference of *P* < 0.001.

In the linear regression model for force at mechanical failure, SBSI was the only significant factor (*p* < 0.001). The 5 mm SBSI was associated with significantly higher load compared to both 7.5 mm (*p* = 0.016) and 10 mm (*p* = 0.001) but not the 3 mm (*p* = 0.20) SBSI (Figure 2). There were no significant differences seen between load at mechanical failure between the 3, 7.5, or 10 mm SBSI (Table 5). SBSI accounted for 10.5% of the variance associated with load at failure or maximum displacement.

**Table 5:** Linear regression for factors associated with load at failure or maximum displacement (*n* = 125).

	**Estimate (Std Error)**	***P*-value**	**LSmeans**	**95% CI**
Intercept	23.78 (2.06)	<0.001	–	–
3 mm bite (Reference)	*–*	*–*	23.78^ab^	19.69–27.86
5 mm bite	3.77 (2.85)	0.2	27.55^a^	23.65–31.44
7.5 mm bite	−4.63 (2.85)	0.1	19.15^b^	15.26–23.05
10 mm bite	−7.23 (2.94)	0.02	16.55^b^	12.39–20.70

*R^2^ = 0.1049. Superscripts with the same letter indicate no significant difference in least squares means (LSmeans) between groups. Superscripts with different letters indicate significantly different LSmeans between groups*.

### Power Calculation

A power analysis using our study data was performed, and the number of samples required to obtain significant differences between groups, using an alpha of 0.05 and power of 0.8 were calculated. Table 6 showed that load per SBSI was the only outcome for which there was a sufficient sample size to determine differences between at least one pair of groups. The other outcomes all showed a lack of power. For instance, in order to fully determine if the cranial, caudal or umbilical regions have different strengths, we would need to have 302 samples in each location group.

**Table 6:** Minimal number of samples per test group to achieve a power of 0.8 and alpha of 0.05.

	**Outcome type**	**Effect size**	**Minimal sample size**
Mechanical failure per site	Dichotomous	0.33	138
Visual failure per site	Dichotomous	1.06	46
Load per bite	Continuous	0.38	21
Load per location	Continuous	0.10	302

## Discussion

### Descriptive

Most samples (92%) underwent mechanical failure (Table 2), regardless of sample location or SBSI. The most common visually identified form of failure was the tissue stretching, or the sutures tearing through the tissue. This is similar to results from a recent study, whereby the majority of feline linea samples failed by muscle or fascial tearing (12). In the previously referenced feline study, their measurement of SBSI started at the edge of the muscle and not at the cut edge of the fascia. In some patients with a wide linea, the fascia will extend beyond this cut edge. The actual width of the linea alba will also differ between cranial and caudal segments of the linea alba. Though it had a much larger sample size, this current study has failed to provide sufficient evidence to support all the findings of the other study.

Suture breakage in this study only occurred in 4 of the 125 samples. This differs from a similar study performed on canine fascia lata in which the polydioxanone suture failed solely by suture breakage (14). That study used 4-0 PDS while the current study used 3-0, therefore suture diameter differences may impact comparison and interpretation of results between the two studies. This could also potentially indicate a difference in the tensile strength of canine and feline fascia.

### Visual Failure

The current study indicates that a larger SBSI is associated with increased odds of visual failure, with 3 mm associated with the lowest odds. This makes sense as a larger distance between bites allows a larger gap to form as the samples are stretched apart. The clinical significance of visual failure is hard to gauge as mechanical failure occurred in many more samples than were seen to have failed (as per Table 2). Not all of the visual failures occurred in the group that had mechanical failures. Clinically, this may confirm a surgeon would have a false sense of incision security based only on visual observation. To the authors' knowledge no previous study has included the visual failure of samples in statistical analysis. In living tissue, the bite sizes that are further from the linea edge are more likely to incorporate non-fascial soft tissue such as fat or muscle, and the sutures cut through these non-fascial tissues when pressure is applied (8). The authors hypothesize that subsequently, slack is created in the sutures and the incisional edges are less likely to remain opposed. Although every effort was made to include only fascial tissue in the bites, a similar effect may have occurred in the cadaver tissue used in this study.

Increase in weight was associated with reduced odds of visual failure regardless of SBSI. Interestingly, body weight was not found to be an important covariate in previous studies performed on equine and canine linea alba closure (4, 15). As cats generally do not have a large range in size within their species relative to other species, this finding was surprising. This may mean that cat body weight plays more of a significant role in linea strength compared to other species and is a possible limitation of this study. A possible explanation could be that higher weight cats had thicker fascial tissue able to withstand higher loads. Fascial thickness was not measured in this study. Without a medical history from the cadavers, it is also possible that some of the cats had underlying metabolic or endocrine disorders which may have contributed to a lower body weight and to decreased tensile strength of their fascial tissue.

### Mechanical Failure

Load was negatively associated with the odds of mechanical failure. That is, for each unit increase in load the odds of mechanical failure decreased. This finding was unexpected as increased load was thought to be associated with greater odds of failure. Since over 90% of all test groups underwent mechanical failure, perhaps the non-mechanical failure group was too small to yield significant difference. The average load at maximum displacement and load at failure were 39.8 and 20.3N, respectively. These values are lower compared to a recent similar study, where the average breaking strength was 58.7N for a 2.5 cm sample for fascia-only closure samples (12). That study used fewer samples compared to the current study, so the difference in load may be explained by differences in the sample population, segment location, and most importantly testing construct. As stated previously, the way they measured their bites differed in that the measurement they used was from the edge of the muscle and did not take into account the linea width that was only fascia which would also differ between cranial and caudal aspects of the abdomen. Rodriguez et al. also indicated that they required a sample size of 20 samples per body wall segment in order to obtain 80% statistical power. Although we were in excess of 20 samples per body wall segment our data showed such a small difference in average load experienced between sample groups that our power calculation indicated a need for 302 samples per location. This could indicate that repairs of this kind are weaker than originally thought. In cadaveric equine linea alba, the breaking strength ranged from 71.4 to 101.4N over a 4.5 cm sample depending on thickness of linea alba (4). Another similar study in cadaveric canine linea alba described the load at failure to be 26.2N over 5 cm segments (15). This further demonstrates the need to assess incisional closure in each species, and that extrapolating data across species may not be appropriate (16).

Location of the sample was not found to be a significant covariate in any of the models. This is different from a previous study performed in horses, which found that samples taken at or close to the umbilicus had the greatest breaking strength (4). It is also different from a recent similar study on feline linea, where the authors found that the linea caudal to the umbilicus sustained the lowest strain to failure (12). The authors of that study hypothesize this may be due to decreasing thickness of the linea in a cranial to caudal direction. In that study however, they calculated the SBSI from the edge of the muscle and not the cut edge of the linea. The linea alba can sometimes be wider in different areas of the abdomen so this may have contributed to the difference. Very few of the samples in this study failed by suture rupture, whereas in another study performed in cadaveric canine fascia lata, failure occurred consistently due to polydioxanone suture rupture (14). That study used 4-0 polydioxanone, however, so this may indicate a difference in fascia tensile strengths between species, fascial areas, or suture size relative to fascial strength. These results have been confirmed by a similar recent study on feline cadaveric linea, where few of the samples failed by suture rupture (12).

### Force at Failure

Load at maximum displacement or failure was only associated with SBSI. It should be noted that SBSI accounted for only 10.5% of the variance in the response variable in this model. However, it is likely that other factors not measured by the current study are important, and as noted by our power analysis a higher number of samples would be recommended. Previous studies indicate that endocrine abnormalities have an effect on collagen and fascia tensile strength (17, 18). As there was no available history on the feline cadavers it is possible that some of the cats were affected by such disorders. Further study is warranted to determine other factors related to force at failure. Bite sizes of 5 mm accommodated significantly more load compared to 7.5 and 10 mm. In another previous study in dogs, maximum wound strength occurred with sutures 5 mm apart and 5 mm from the incision (15). In a recent similar study in cats, there was no statistical difference in breaking strength when comparing 5 mm to 2 mm SBSI even though in the conclusion they support using a 2 mm SBSI (12). It has also been suggested that a small stitch interval combined with suture bite size is the most resistant to suture pullout, with the ideal stitch interval of 5 mm apart (6). This supports that 5 mm SBSI may be the ideal distance in closing cat linea alba. This study used 5 mm both for the distance from the edge of the linea and from other suture bites so it is possible that a different stitch interval may yield different results. This is supported in the human field where the risk of hernia is lower when the suture length to wound length (SL:WL) ratio is >4 (16, 19–21). This means that the length of suture material that ends up being used to close an incision is 4 times longer than the length of the incision. We did not measure the actual SL:WL ratio in this study. Future studies would need to be performed to investigate the 5 mm bite size with different stitch interval sizes and the SL:WL ratio would need to be measured. By decreasing the stitch interval size (placing stitches closer together), the SL:WL ratio is increased.

Frozen samples were used in this study as the biomechanical properties of collagenous tissues are maintained after multiple freeze-thaw cycles (22). Frozen samples were also chosen over fresh cadavers to avoid biomechanical changes from decomposition, to conform with biosecurity protocol at the Zymetrix laboratory, and because there was greater accessibility to frozen cadavers. This is a limitation of our study.

There is a paucity of literature on feline fascial closure so we are limited to comparisons with literature on other species.

## Conclusion

The results of this study indicated that 5 mm SBSI offered the greatest wound load to failure compared to 7.5 or 10 mm in thawed abdominal feline cadaveric tissue. However, future investigation is needed to compare this to 3 mm SBSI.

## Data Availability Statement

The datasets generated for this study are available on request to the corresponding author.

## Author Contributions

AB performed the background research, helped devise the methods, performed the experiment, did the statistical analysis, and wrote the original manuscript draft. AA guided the development of the testing protocol, supervised the experiment, has been involved with major editing of the manuscript. RA helped with design of the experiment, and edit the manuscript. GK helped consult with design and statistical analysis, helped AB perform the statistical analysis, and helped edit the manuscript for submission.

### Conflict of Interest

The authors declare that the research was conducted in the absence of any commercial or financial relationships that could be construed as a potential conflict of interest.

## Supplementary Material

Supplementary Figure 1 Picture of the Electroforce 3200 at maximum displacement (12 mm) with a tissue sample in place (1 cm SBSI).
