# Supplementary figures and images for: Optimal Suture Bite Size for Closure of Feline Linea Alba—A Cadaveric Study
Source: Front Vet Sci. 2019 Dec 10;6:441. doi: 10.3389/fvets.2019.00441 (PMC6914685; doi:10.3389/fvets.2019.00441)

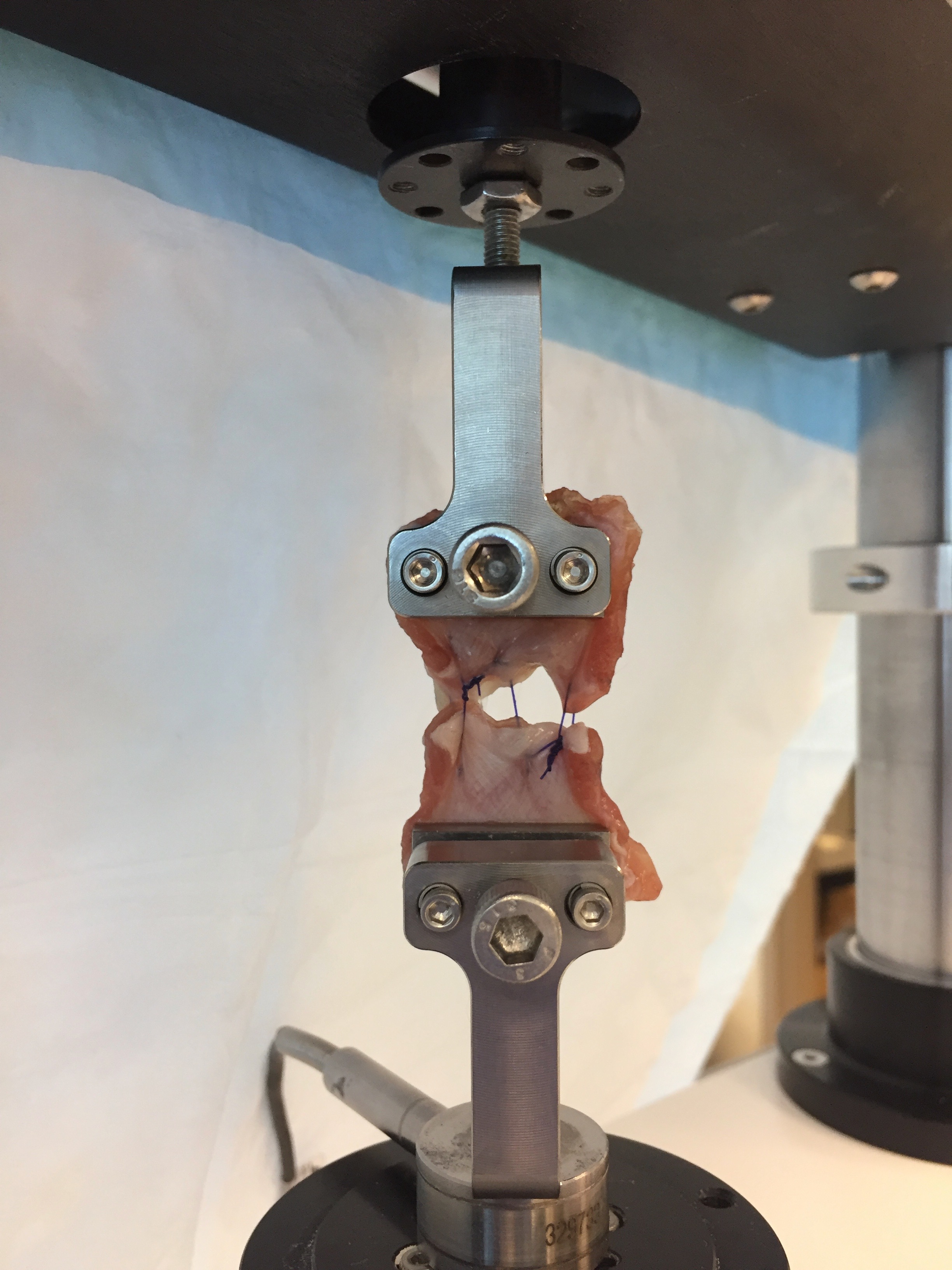

Supplement: Supplementary Figure 1 — Picture of the Electroforce 3200 at maximum displacement (12 mm) with a tissue sample in place (1 cm SBSI). [file Image_1.JPEG]
